# Simulation-assisted machine learning

**DOI:** 10.1093/bioinformatics/btz199

**Published:** 2019-03-23

**Authors:** Timo M Deist, Andrew Patti, Zhaoqi Wang, David Krane, Taylor Sorenson, David Craft

**Affiliations:** 1 Department of Radiation Oncology, Massachusetts General Hospital, Harvard Medical School, Boston, Massachusetts, USA; 2 The D-Lab: Decision Support for Precision Medicine, GROW - School for Oncology and Developmental Biology, Maastricht University Medical Centre, Maastricht ER, The Netherlands

## Abstract

**Motivation:**

In a predictive modeling setting, if sufficient details of the system behavior are known, one can build and use a simulation for making predictions. When sufficient system details are not known, one typically turns to machine learning, which builds a black-box model of the system using a large dataset of input sample features and outputs. We consider a setting which is between these two extremes: some details of the system mechanics are known but not enough for creating simulations that can be used to make high quality predictions. In this context we propose using approximate simulations to build a kernel for use in kernelized machine learning methods, such as support vector machines. The results of multiple simulations (under various uncertainty scenarios) are used to compute similarity measures between every pair of samples: sample pairs are given a high similarity score if they behave similarly under a wide range of simulation parameters. These similarity values, rather than the original high dimensional feature data, are used to build the kernel.

**Results:**

We demonstrate and explore the simulation-based kernel (SimKern) concept using four synthetic complex systems—three biologically inspired models and one network flow optimization model. We show that, when the number of training samples is small compared to the number of features, the SimKern approach dominates over no-prior-knowledge methods. This approach should be applicable in all disciplines where predictive models are sought and informative yet approximate simulations are available.

**Availability and implementation:**

The Python SimKern software, the demonstration models (in MATLAB, R), and the datasets are available at https://github.com/davidcraft/SimKern.

**Supplementary information:**

Supplementary data are available at *Bioinformatics* online.

## 1 Introduction and motivation

There are two general approaches to computationally predicting the behavior of complex systems, simulation and machine learning (ML). Simulation is the preferred method if the dynamics of the system being studied are known in sufficient detail that one can simulate its behavior with high fidelity and map the system behavior to the output to be predicted. ML is valuable when the system defies accurate simulation but enough data exist to train a general black-box machine learner, which could be anything from a linear regression or classification model to a neural network. In this work, we propose a technique to combine simulation and ML in order to leverage the best aspects of both and produce a system that is superior to either technique alone.

Our motivation is personalized medicine: how do we assign the right drug or drug combination to cancer patients? Across cultures and history, physicians prescribe medicines and interventions based on how the patient is predicted to respond. Currently these choices are made based on established patient-classification protocols, physician judgment, clinical trial eligibility and occasionally limited genomic profiling of the patient. All of these approaches, in one way or another, attempt to partition patients into groups based on some notion of similarity.

Genomics is especially relevant for computing the similarity between two cancer patients since cancer is associated with alterations to the DNA, which in turn causes the dysregulation of cellular behavior (Balmain *et al.*, 2003). Bioinformatic analysis has revealed that there is heterogeneity both within a patient tumor and across tumors; no two tumors are the same genomically (Felipe De Sousa *et al.*, 2013; Fisher *et al.*, 2013). Although in a small fraction of cases specific genetic conditions are used to guide therapy choices, for example, breast (commonly amplified gene: HER2), melanoma (BRAF mutation), lung (EML4-ALK fusion) and head-and-neck (HPV status for radiation dose de-escalation; see Mirghani and Blanchard, 2017), there remains a large variability in patient responses to these and other treatments, likely due to the fact that patients will usually have tens or hundreds of mutations and gene copy number variations, chromosomal structural rearrangements, not to mention a distinct germline genetic state (Hauser *et al.*, 2018), human leukocyte antigen (HLA) type (Chowell *et al.*, 2018), tumor epigenetic DNA modifications, microbiome, and comorbidity set. Even amidst this heterogeneity, the notion of patient similarity—although currently not deeply understood due to the complexities of cancer biology—is appealing both conceptually and for its value in the ML setting.

Simulating a drug is a task that far exceeds our current scientific capacity: it enters the patient, either intravenously or orally, and winds its way to the cancer cells, where it either influences the cancer cell via receptors on the cell membrane or penetrates into the cell and affects signaling pathways, cell metabolism, DNA repair, apoptosis, or some combination of these and other modules. Nevertheless, a vast amount of knowledge of cellular processes, residing in molecular biology textbooks and millions of scientific papers, has been accrued over the past century and it seems worthwhile to attempt to use that information, if unclear how. Most machine learning research efforts in the personalized medicine realm take a pure data approach. Given the complexity of patient biology and cancer, this approach will require vast amounts of high quality patient data that is suitably standardized for algorithmic processing.

**Fig. 1.**
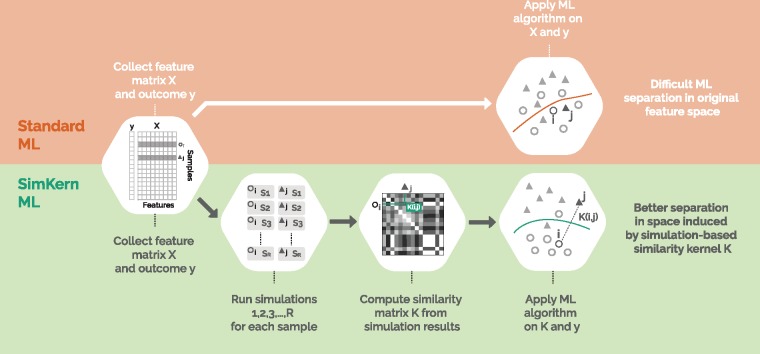
Workflow comparison of Standard ML and SimKern ML. The feature matrix *X* and outcome data *y* are given (in this paper, we generate such ‘ground truth’ datasets by simulating complex systems, a step which is not shown in this figure). Traditional feature-based ML is depicted in the upper orange part. SimKern, the simulation-based method, pre-processes the dataset by sending each sample through a number of approximate simulations. Each sample pair is given a similarity score based on how closely they behave under the various simulations (see Fig. 2 and Section 2.2 for more details). This information is stored in a kernel matrix *K*, where *K*(*i*, *j*) measures the similarity between samples *i* and *j*. Note that K(i,i)=1 and 0≤K(i,j)≤1. Useful SimKern simulations yield a kernel *K* that improves the downstream machine learning performance

With this drug sensitivity prediction problem as our backdrop, we develop a method to combine approximate simulations with ML and demonstrate using *in silico* experiments that a judicious combination can yield better predictions than either technique alone. The basic idea is a division of labor: coarse and approximate simulations are used to compute similarity measures, and these similarity measures are then used by the ML algorithm to build a predictive model, called SimKern ML (Fig. 1). At this point in time, although vast details of cellular biology are known, we are not in a position to simulate with any fidelity complete cellular or *in vivo* cancerous processes. However, herein we present demonstrations that one could combine simulation results into machine learning and improve the overall predictive capability, a technique which may play a role in future drug recommendation systems.

## 2 Materials and methods

Our method is centered on kernelized ML. Rather than feature vectors (a list of attributes for each sample), kernelized learning requires only a similarity score between pairs of samples. For training, one needs the outcome of each training sample and a measurement of the similarity between all pairs of training samples. For predicting the outcome of a new sample, one needs to provide the similarity of that sample to each training sample. It is well known in ML that good similarity measures, which come from expert domain knowledge, result in better ML performance (Schölkopf *et al.*, 2004). We assume that we can formulate a simulation of each sample’s behavior based on its known individual characteristics (i.e. features). We also assume that we do not know exactly how to simulate the systems, so rather than a single simulation we have a family (possibly parametrized by real numbers, and thus infinite) of plausible simulations. Two samples are given a high similarity score if they behave similarly across a wide range of simulations.

We begin with a brief description of the four models we use to demonstrate and analyze the performance of SimKern. By describing these models, the reader has in mind a more concrete context with which to frame the SimKern development.

### 2.1 Brief model descriptions

We investigate four models: radiation impact on cells, flowering time in plants, a Boolean cancer model and a network flow optimization problem. Full details and model implementation notes are given in the Supplementary Material.

For each model we begin by generating a dataset of *N* samples, each sample *i* is described by a feature vector *x_i_* of length *p* and a response *y_i_*, using the ground truth simulation (see Supplementary Fig. S1). This produces an *N *×* p* feature matrix *X* and a response vector *y* of length *N*. This ground truth simulation (referred to as SIM0 in the code repository) is not part of our kernelized learning method, but the datasets created are needed to demonstrate the simulation-based kernel ML method. This ground truth simulation step is further described in the Supplementary Material. In an actual application of SimKern, this artificial data creation step would not be used.

The **radiation cancer cell death model** is a set of ordinary differential equations (ODEs) which represents a simplified view of the biochemical processes that happen after a cancer cell is hit by radiation. The core of the model involves the DNA damage response regulated by the phosphorylation of ATM and subsequent p53 tetramerization (Eliaš *et al.*, 2014). We have added cell cycle arrest terms, a DNA repair process, and apoptosis modules in order to capture the idea that cellular response to DNA damage involves the combined dynamics of these various processes. The model, which is depicted as a network graph, is displayed in Supplementary Figure S3, and consists of 34 ODEs. The rate parameters were not tuned to realistic values (except for the ones from the original p53 core network, where we used the values provided by the authors; see Eliaš *et al.*, 2014). Instead, values were manually chosen such that the family of samples created had representatives in each of the four output classes: apoptosis, repaired and cycling, mitotic catastrophe, and quiescence. A population of distinct cell types is formed by varying 33 of the ODE rate constants and the mutation status of six genes (ARF, BAX, SIAH, Reprimo, p53 and APAF1), for a feature vector length of 39. The SimKern simulation uses the same underlying model as the original ODE model with two key differences: 87 of the ODE parameters are marked as uncertain and given Gaussian probability distributions around their true values, and the simulation outputs the time dynamics of the ODEs rather than a classification.

The **flowering time model** is a set of six ODEs that simulate the gene regulatory network governing the flowering of the Arabidopsis plant (Valentim et al., 2015), and yields a regression problem. Nineteen mutants are modeled and experimentally validated by the authors. We use those 19 mutational states as well as 34 additional perturbations on the rate parameters to create a varied ground truth sample set. The output of the model is the time to flowering which, following the authors, is set to the time at which the protein AP1 exceeds a particular threshold. For the SimKern model we assume the same model but with uncertainty about the rate parameters. The SimKern simulation output is the time dynamics of the six ODEs.

The **Boolean cancer model** is a discrete dynamical system of cancer cellular states (Cohen *et al.*, 2015). Based on the steady state of the system, a sample is labelled as one of three categories: apoptotic, metastasizing, or other. There are no rate parameters since this is a Boolean model. We use the initial state vector (the on/off status of the 32 nodes in the network) as well as mutations of five of the genes (p53, AKT1, AKT2, NICD and TGFβ) to create a varied sample population with 37 features. In the SimKern simulation, we use a reduced version of the model provided in the original publication. It is unclear how to map the initial conditions from the full model to the initial conditions of the modularly-reduced model, so for all of the modules we randomly choose the mapping, which gives rise to the uncertainty for the SimKern simulations. The output from the SimKern model (i.e. the data used to form the similarity matrix) is the same classification as from the ground truth model.

The **network flow model** is an optimization problem rather than a simulation. It falls into a subclass of linear optimization models called network flows which are used in a wide range of applications including production scheduling and transportation logistics (Bertsimas and Tsitsiklis, 1997). The network flow model takes arc costs as inputs, which are the costs of sending a unit of flow through a certain arc in the network. The model then simulates the optimal path of flow along arcs of a directed graph that minimizes the total arc cost along the path. The network is designed in layers and is such that the flow will pass through exactly one of the three arcs in the final layer, which gives us a classification problem (see Supplementary Fig. S2). Changes in arc costs, which represent the features in this model, can lead to changes in the routing of the optimal flow. For the ground truth dataset, we generate samples by varying 12 out of the 80 arc costs. We build two separate SimKern simulations: the better simulation perturbs 23 arc costs, including the 12 costs that were varied to make the ground truth dataset, resulting in a less noisy kernel. The worse simulation varies 21 additional arc costs resulting in a noisier kernel.

### 2.2 SimKern simulation—similarity matrix generation

**Fig. 2.**
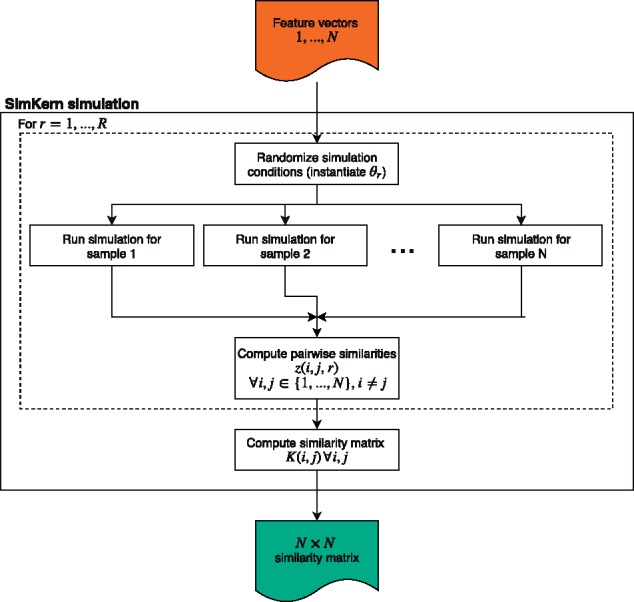
Creation of the similarity matrix for use downstream in the machine learning

Users must define a model (currently supported languages for the simulation modeling are MATLAB, Octave, and R) which simulates a sample. This simulation procedure, called SIM1 in the Python codebase, is used to generate the sample similarity kernel matrix and would be the starting point in an actual application of SimKern. Figure 2 illustrates the SimKern simulation process control.

We assume that there are parameters in this simulation model that we are uncertain about. Let θ be a vector of these uncertain parameters. We assume we have a random variable description of each of these parameters, which can be very general. For example, a parameter could take the value of 0 or 1 if we have two ways of modeling a particular interaction. Then, in the simulation, depending on how that random variable gets instantiated, the code uses one of the two parameter values. Alternatively, we might be uncertain about the value of a rate constant, in which case we could use a Gaussian random variable with a specified mean and standard deviation. We assume independence of the random variables θ, but one could also assume a covariance structure.

Each sample i=1…N is characterized by a feature vector *x_i_*, which constitutes sample-specific information that we use to perform the simulations; *x_i_* could be for example a genomic description of patient *i*. For r=1…R, where *R* is the number of trials to run, we instantiate a parameter vector, θ_*r*_. These parameters as well as the sample data *x_i_* are used to run simulation (*i*, *r*).

Let $S(x_{i},\theta_{r})$ (or shorthand, *S_ir_*) be the simulation output for sample *i* with uncertainty parameters equal to θ_r_. Note that these outputs $S(x_{i},\theta_{r})$ can be scalars, a classification category, vectors, or any other object. There is no need for these outputs to be the same as what we are trying to predict, *y_i_*. We simply assume that given two such outputs, say *S_ir_* and *S_jr_* for samples *i* and *j*, we have a way to measure the similarity between them. Let this similarity be given by z(i,j,r). We leave it up to the user to define this function in general (a concrete procedure, for simulations using ODEs, is given in the Supplementary Material).

Finally, the similarity *K*(*i*, *j*) between two samples *i* and *j* is the average similarity across the *R* simulation runs

$$K(i,j)=(1/R)\sum_{r=1}^{R}z(i,j,r).$$

The above SimKern kernel matrix generation procedure is implemented in Python and is fully described in the Supplementary Material.

### 2.3 Machine learning comparisons procedure

Figure 1 shows a schematic of the differences in the data processing and machine learning steps for Standard ML and SimKern ML. We compare standard feature-based ML algorithms [orange/top: linear support vector machine (SVM), radial basis function (RBF) SVM and random forest (RF)] with simulation kernel based methods (green/bottom: kernelized SVM and kernelized RF). We also include results for one-nearest neighbor (NN) and kernelized one-nearest neighbor (SimKern NN). As NN-type algorithms are arguably the simplest non-trivial ML algorithms, including these algorithms allows us to understand the distinct contributions of ML algorithm sophistication and simulation-based kernels.

Since we can generate as many samples as we wish, we train the models and tune the hyperparameters on training and validation datasets which are distinct from the final testing set on which we compute prediction performance metrics (see Section 2.4). The ground truth simulation generates one dataset which is split into three parts (train/validation/test) using the standard proportions 50%/25%/25% (Hastie *et al.*, 2009, p. 222). SVM (Ben-Hur and Weston, 2010) and NN algorithms are dependent on feature scaling, therefore features are standardized to the interval [0,1] by subtracting the minimum value and scaling by the range. Categorical features are dummy-coded for SVM and NN algorithms. Each ML algorithm is trained on the training data for many hyperparameter configurations and the configuration with the best fit on the validation data is selected. The model given the selected configuration is applied on the test set to compute the performance metrics. See Alg. 1 in the Supplementary Material for the details of training, hyperparameter tuning, and testing procedures.

To investigate the performance of simulation-based kernels in scenarios with less data for training, we consider five scenarios in which we train the algorithms on subsamples comprising *s*_1_, *s*_2_,…, *s*_5_ of the training data. The subsampling percentages are chosen differently per model to highlight the interesting regions of curves that display the performance versus training set size. Supplementary Table S3 reports the subsampling percentages per model.

### 2.4 Performance metrics

For each of the simulation models, we estimate the generalization performance of an ML algorithm in test data, i.e. data unused for model training, as performance estimates on training data are of little practical value (Hastie *et al.*, 2009, p. 230). The learning tasks per model are either classification or regression. For classification, we consider prediction accuracy, which is defined as

$$\text{Accuracy}=\frac{\text{true}\;\text{classification}\;\text{count}}{\text{total}\;\text{number}\;\text{of}\;\text{samples}}=\frac{\text{TP}+\text{TN}}{\text{TP}+\text{TN}+\text{FP}+\text{FN}},$$

where TP, TN, FP and FN are the counts of true positives, true negatives, false positives, and false negatives, respectively. For regression, we consider the coefficient of determination *R*^2^, which is defined as

$$R^{2}=1-\frac{\text{sum}\;\text{of}\;\text{squared}\;\text{prediction}\;\text{error}}{\text{sum}\;\text{of}\;\text{squares}}=1-\frac{\sum_{i}{\left({\hat{y}}_{i}-y_{i}\right)}^{2}}{\sum_{i}{\left(\bar{y}-y_{i}\right)}^{2}},$$

where *y_i_* is the outcome for sample *i*, ${\hat{y}}_{i}$ is the predicted outcome for sample *i* and $\bar{y}$ is the sample mean of the outcome. To attain a reliable estimate of the generalization performance, we consider the average test data performance in ten repetitions of a train/validation/test analysis, i.e. repeating training and hyperparameter tuning each time.

### 2.5 Standard ML versus SimKern ML comparison

For each model, we produce a box plot and/or a line plot that show algorithm performance versus training dataset size for the various ML algorithms in both algorithm groups, Standard ML and SimKern ML.

- Box plots display results for each algorithm separately for the Standard ML (linear SVM, RBF SVM, RF, NN) and SimKern ML algorithms (SimKern SVM, SimKern RF, SimKern NN). The horizontal lines indicate the sample median, the boxes are placed between the first and third quartile (*q*_1_, *q*_3_). Outliers are defined as samples outside $\left[q_{1}-1.5(q_{3}-q_{1}),q_{3}+1.5(q_{3}-q_{1})\right]$ and are indicated by crosses.
- Line plots further condense the findings by displaying the median performance metric of the best performing Standard ML and SimKern ML algorithms, excluding NN algorithms in both cases. The best performing algorithm is defined as the algorithm that most frequently produces the highest median performance metric over all five training dataset subsamples. Lines are interpolated for visual guidance.

### 2.6 Sensitivity analysis

To investigate possible factors affecting the SimKern algorithms’ prediction performance, we run the following sensitivity analyses:

**Varying prior knowledge**

- Radiation model: we examine the results for two kernels which represent different levels of prior knowledge. Both cases utilize the same SimKern simulation, but the higher quality kernel uses the dynamics of only the compartments of the ODE set that are used in the classification of the samples in the initial ground truth simulation. The lower quality kernel uses all ODE equations, therefore not emphasizing the most important ones (Ferranti *et al.*, 2017).

**Varying simulation parameter noise/bias**

ii. Network flow model: we generate two kernels for the network flow model. These kernels differ in the number of arc costs that are perturbed and the size of the perturbations (full details in Supplementary Material).
iii. Flowering time model: along with the model that generates the baseline kernel, we study one less noisy, one noisier, and one biased version of the SimKern simulation. The baseline SimKern simulation uses multiplicative Gaussian noise on 34 of the rate parameters, using a mean of 1 and a standard deviation of 0.2. The less noisy model uses stdev=0.1 and the noisier model uses stdev=0.4. For a more radical, and non-centered, departure from the true rate parameters, we also run a model where we multiply each of the same 34 rate parameters with a random variable chosen uniformly from the discrete set {0.01, 1, 5, 10}.

**Varying the number of simulation trials, *R***

iv. Network flow model: we analyze the effect of additional simulation trials on the prediction performance. We compare the prediction performance of SimKern algorithms when using a similarity kernel based on *R* = 3 simulation trials to the final kernel based on *R* = 10 trials. Furthermore, we track the convergence of the kernel matrix over *R* = 10 trials.

## 3 Results

The general theme that emerges is that, for small training dataset sizes, the methods using the SimKern kernel outperform the Standard ML methods. For larger training sizes, however, the standard methods either approach the SimKern methods or exceed them, depending on the quality of the kernel.

**Fig. 3.**
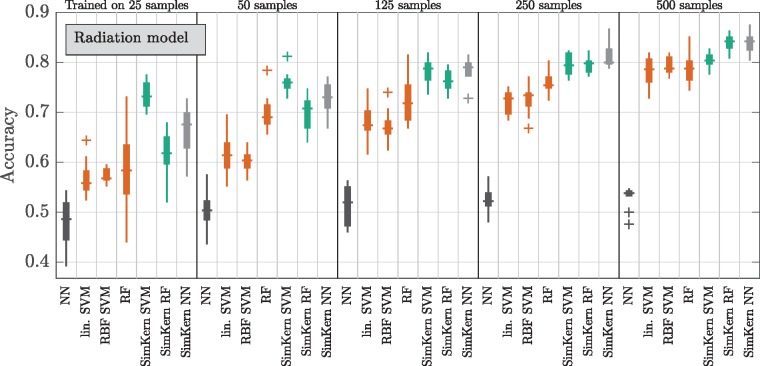
Machine learning results for the radiation cancer model

**Fig. 4.**
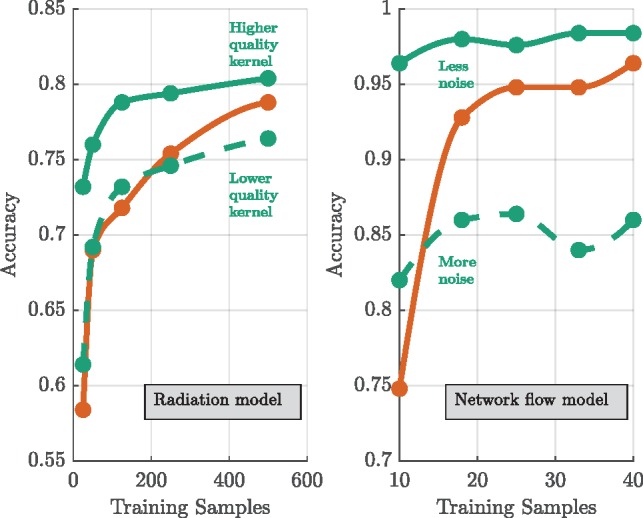
Varying prior knowledge experiments for the radiation model (left) and varying parameter noise experiments for the network flow model (right). Performance metrics of SimKern ML based on simulations with less and more prior knowledge (green) and Standard ML (orange). For each line, the best performing algorithm of SimKern ML or Standard ML is selected (see Section 2.5). Note, the waviness of the less noise case for the network flow model is an artifact of how the data from the box plots was converted into a line plot; the full data, Supplementary Figure S7, reveals a flat relationship

For the radiation model, we see exactly this general pattern (Fig. 3). For small training sizes (up to 50 samples), the SVM with the SimKern kernel dominates. We can attribute much of the performance gain to the similarity kernel itself given that the NN algorithm using the same similarity kernel also dominates over the no-prior-knowledge methods for all training sizes shown. The increase in accuracy by the Standard ML algorithms does not yet show signs of saturation by 500 training samples. These box plots are summarized by line plots in Fig. 4 (left), which also displays the results of the lower quality SimKern kernel, which was made with the same simulations but without focusing on the most relevant ODEs for the kernel matrix computation.

**Fig. 5.**
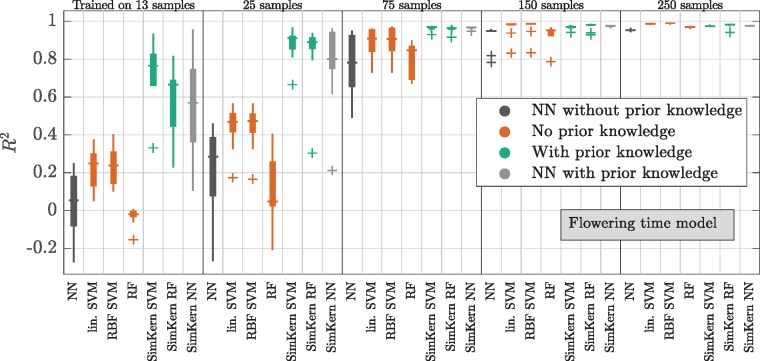
Machine learning results for the flowering time model

The results of the flowering time model, which also display the clear dominance of SimKern learning for small training dataset sizes, show a trend of decreasing variance in predictive performance with increasing training sizes (Fig. 5). SimKern learning is strongly dominant up to 75 training samples, after which the two learning styles converge to $R^{2}\approx 1$. Another view of the improvement offered by the SimKern method for small training size set sizes is shown by plotting the predicted flowering times versus the actual flowering times, Supplementary Figure S9.

**Fig. 6.**
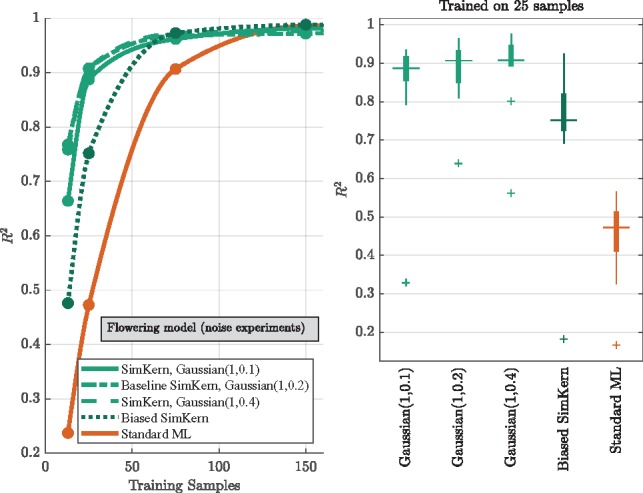
Varying simulation parameter noise/bias experiments for the flowering time model. SimKern ML based on simulations with varying parameter noise (green), with parameter bias (dark green), and Standard ML (orange). Left: performance metrics of SimKern ML (green) and Standard ML (orange) trained on up to 150 samples. For each line, the best performing algorithm of SimKern ML or Standard ML is selected (see Section 2.5). Right: performance metrics box plots for the 25 training sample case

The sensitivity results obtained by increasing the variance of the (centered) Gaussian noise that was applied to the flowering model’s rate parameters display a robustness to these deviations (Fig. 6, upper green curves and Gaussian box plots). However, the non-centered noise perturbation analysis shows a clear drop in ML accuracy (Fig. 6, dark green dotted line and dark green box plot). With enough training data all SimKern kernels, including the ones with heavy noise, achieve an *R*^2^ above 0.95. We call such kernels *sufficient*.

**Fig. 7.**
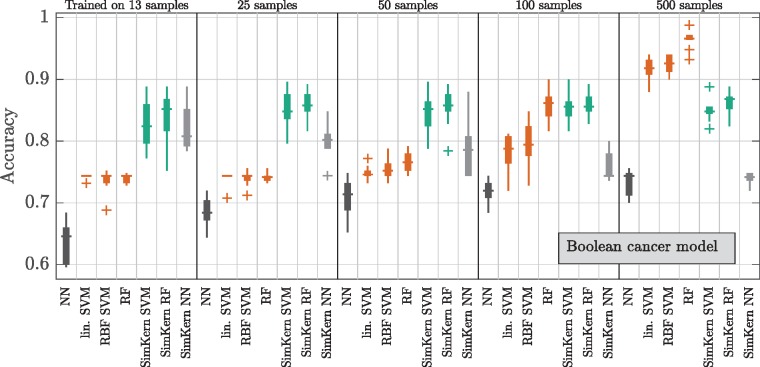
Machine learning results for the Boolean cancer model

In contrast, the Boolean cancer model kernel is based on a model reduction with additional uncertainty and produces what we call a *biased* kernel. There, the SimKern approach produces an accuracy that initially dominates but quickly plateaus to around 85% and is overtaken by no-prior-knowledge methods when more training data is available (Fig. 7). The fact that the kernel learning barely improves with additional data implies that the feature space induced by the simulation kernel is simple enough to be learned by a small amount of samples (Bottou *et al.*, 1994). The kernelized NN method gets worse with more samples, and in general is worse than the other SimKern algorithms, which indicates that the space induced by the biased kernel is less cleanly separable compared to the flowering model case. Above 100 training samples, the no-prior-knowledge RF method is the superior technique.

For the network flow problem, we evaluate two separate kernels (Fig. 4, right) based on different levels of noise in the SimKern simulation: the kernel based on a less noisy SimKern simulation dominates throughout, but even the kernel based on a noisier SimKern simulation is still useful in the very small training set size range. It is doubtful whether one can make general statements about how good a simulation needs to be in order to yield a useful kernel. However, the intuition that the simulations need only discover the similarity of samples, while not necessarily providing accurate (hence directly useful) simulation results, is described in Supplementary Figure S8.

When comparing the individual Standard ML algorithms to the SimKern ML algorithms based on the noisier SimKern simulation (Supplementary Fig. S7), Standard RF eventually dominates. When comparing algorithms within the Standard ML group, RF is the dominant Standard ML algorithm for the network flow model (Supplementary Fig. S7) as well as for the Boolean cancer model (Fig. 7). For these models, the dominance of RF is likely related to the discrete characteristics of the underlying models.

**Fig. 8.**
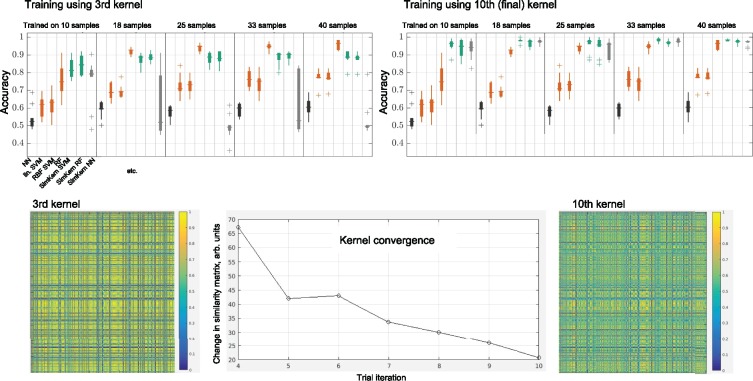
Varying simulation trials experiments for the network flow model. The upper two box plots compare the learning accuracy for a kernel from the third of *R *=* *10 trials versus the final kernel. The kernels themselves are displayed with the same color scale below, and centered at bottom displays the convergence of the kernel (measured using the Frobenius matrix norm) over the ten trials

The quality of a simulation-generated kernel also depends on the number of trials *R* that are used to compute the kernel. Figure 8 displays both the convergence of the kernel (bottom) and the improved learning accuracy from the further converged kernel (top), for the less noisy network flow case. We see that the earliest kernel written, kernel three (we chose to not determine similarity kernels below *R *=* *3), performs noticeably worse than the final kernel. We can also visually observe the differences in the kernels by plotting the 500 × 500 kernels (Fig. 8, bottom left and right). The kernel convergence plot is obtained by taking the Frobenius norms of the difference of the kernel matrices of iteration *i* – 1 and *i*, until i=R(=10).

## 4 Discussion

We introduce simulation as a pre-processing step in a machine learning pipeline, in particular, as a way to include expert prior knowledge. One can consider simulation as a technique which regularizes data or as a specialized feature extraction method. In either view, the SimKern methodology offers a decomposition of an overall ML task into two steps: similarity computation followed by predictive modeling using the pairwise similarities. This decomposition highlights that to improve the performance of an ML model one can direct efforts into determining better similarity scores between all samples. This is in contrast to the more commonly heard call for ‘more data’ to achieve better ML results. Of course, more samples are always desirable, but here we show that, particularly in limited data settings, sizable performance gains can come from high quality similarity scores.

The decomposition of simulation and machine learning steps also points out their individual contributions. The simulation-based kernel structures the space in which the samples live (or more technically, the dual of the space; see Kung, 2014), and ML finds the patterns in this simplified space. We see that in order to improve machine learning performance we can either improve the kernel or increase the number of samples to better populate the space. For the cases shown here, custom similarity measures show large improvements especially in limited data settings (up to a 20% increase in classification accuracy and a 2.5 fold increase in *R*^2^, depending on the case and the amount of training data used). One could also use the output of the simulations as features for machine learning rather than the additional kernelization step that we employed. Using the simulation outputs directly is related to the field of model output statistics from weather forecasting, where low level data from primary simulations are used as inputs to a multiple regression model which outputs human-friendly weather predictions (Glahn and Lowry, 1972). In our case, we opted for kernelizing the simulation outputs to highlight the fundamental concept of similarity and because a similarity computation is natural when the output of the simulations is a set of time varying entities, e.g. in the case of ODEs.

Similar in spirit to SimKern, although differing in details, combining simulation and machine learning has been used in physics to predict object behaviour (Lerer *et al.*, 2016; Wu *et al.*, 2015). Simulation results are used to train networks to ‘learn’ the physics. Varying the simulation conditions during training, called *domain randomization*, is used to improve model generalization (Tobin *et al.*, 2017). Inversely to the SimKern approach to exploit simulation to enhance ML algorithms, machine learning is also used to correct the inputs to physics simulations (Duraisamy *et al.*, 2015), an idea which is also pursued in the context of traffic prediction (Othman and Tan, 2018).

A novel potential application of the SimKern methodology, one that the authors are currently investigating, involves the prediction of peptides (chains of approximately nine amino acids) binding to a given HLA class 1 allele. Current technologies (e.g. Nielsen *et al.*, 2007) predict if a given peptide will bind to a given HLA allele using properties of the amino acids but without using 3D details of the chemical structure of the peptide or information on the structural binding of the peptide and HLA molecule. Computational predictions of binding are considered too difficult at the present time due to the sensitivity of the structural conformations to the detailed chemistry of peptides and the non-covalent interactions (Kar *et al.*, 2018). Nevertheless, simulations could be used to generate similarity scores between peptides, and then the supervised binding data can be used to train a kernelized classification algorithm.

Finally, the use of a SimKern kernel need not be an all-or-nothing decision, since two or more kernels can be combined to yield a single kernel. This allows one to explore the combination of ‘standard’ kernelized learning (using uninformed kernels such as linear or RBF) with a SimKern kernel. In the case of a weighted linear sum as the method of kernel combining, one can optimize the weighting vector as part of the training procedure (Schölkopf *et al.*, 2004). Combining kernels allows one to mix traditional feature-based machine learning (which we called Standard ML above) with prior knowledge similarity matrix-based learning.

## 5 Conclusions

It remains to be seen which approaches will be the most fruitful as we make our way towards personalized cancer medicine. Direct testing of chemotherapeutic agents on biopsied patient tissues is a straightforward and promising ‘hardware-based’ approach (Montero *et al.*, 2015). In the machine learning realm, expert feature selection may turn out to be more feasible than the simulation-based kernel methods described in this report. A key question is: can we make simulation-based kernels that—although almost certainly biased—will still be useful (see, e.g. Fig. 4)? Progress in detailed biological simulation, such as the full simulation of the cell cycle of the bacterium *Mycoplasma genitalium* (Karr *et al.*, 2012), the OpenWorm project (Szigeti *et al.*, 2014), and integrated cancer signaling pathways for predicting proliferation and cell death (Bouhaddou *et al.*, 2017) offer some encouragement, but cancer influences human biology at all levels, from minute phosphorylations to immune system rewiring. It is thus by no means clear if we are close to simulations that can be useful in this context. However, the magnitude of the problem—both in economic terms and for the number of future patients at stake—suggests pressing forward on all fronts that display conceptual promise.

*Conflict of Interest*: none declared.

## Supplementary Material

btz199_Supplementary_Data
