# Supplementary material for: Simulation-assisted machine learning
Source: Bioinformatics. 2019 Mar 23;35(20):4072–80. doi: 10.1093/bioinformatics/btz199 (PMC6792064; doi:10.1093/bioinformatics/btz199)
Supplement: btz199_Supplementary_Data [file btz199_supplementary_data.pdf]

Supplementary Information: Simulation assisted machine learning

Timo M. Deist[1,2,*], Andrew Patti[1,*], Zhaoqi Wang[1], David Krane[1], Taylor Sorenson[1], David Craft[1,†]

[1]Department of Radiation Oncology, Massachusetts General Hospital, Harvard Medical School

[2]The D-Lab: Decision Support for Precision Medicine, GROW - School for Oncology and Developmental Biology, Maastricht University Medical Centre

* These authors contributed equally to this work.

†To whom correspondence should be addressed; E-mail: dcraft@broadinstitute.org.

Contents

1 Ground truth and SimKern simulations **2**

1.1 Ground truth data generation procedure: SIM0 2

1.2 Similarity kernel generation: SIM1 5

1.2.1 Similarity as measured by closeness of ODE solutions 5

2 Machine learning details **6**

2.1 Machine learning algorithm comparisons procedure 6

2.1.1 Stratification 8

2.1.2 Hyperparameter tuning 8

2.2 Machine learning algorithms 9

3 Model descriptions **10**

3.1 Radiation model 11

3.1.1 Additional modeling notes 16

3.2 Flowering model 17

3.3 Boolean cancer model 17

3.4 Network flow optimization model 18

4 Supplementary results **20**

5 A word on the "kernel trick" **24**

1 Ground truth and SimKern simulations

The simulation framework, which handles the generation of ground truth data as well as the SimKern module which performs the simulations and computes the similarity matrix, is written in Python, and supports simulation models written in MATLAB, Octave, and R. It uses text file communication so it could be easily adapted to simulations written in other languages. The Python package, SimKern, is available at github: `https://github.com/davidcraft/SimKern`. *We refer to the ground truth simulation as SIM0 and the SimKern simulations as SIM1. This naming convention is also reflected in the Python code base.*

The various code modules are summarized in Table S1.

1.1 Ground truth data generation procedure: SIM0

A simulation model file used to create a ground truth dataset has the suffix .t. A file used to create the SimKern family of simulations is suffixed with .u (see next section). These model files are in the language of the system used to run the simulations and have

Name	Functionality	Requires	Language
Groundtruth dataset generation (“SIM0”)	Generates datasets (features and known outcomes) with user-selected number of samples	The simulation (“SIM0”) model (*.t file)	Python (simulation models though are in Matlab, octave, or R)
SimKern (“SIM1”)	Handle running families of simulations, aggregating results and forming the similarity kernel	Feature vectors that are used to simulate each feature (“genome key” files), and the master *.u file that contains the stochasticity information $\boldsymbol{\theta}$	Python (as above)
Machine Learning Comparison	Tune and train models with all machine learning algorithms on various dataset sizes for comparison	Sample features for standard machine learning, sample similarity matrix for kernelized learning, and sample outcomes	Matlab (also available in the SimKern python repository, but Matlab version used for the results in the paper)

Table S1: Code module descriptions.

entities that are set off by dollar signs. These entities are the parameters to vary from one sample to the next, for the ground truth dataset generation, or from one trial to the next, for the SimKern generation.

As an example, if different samples may have different values for a rate parameter called k_1, a line in the simulation file could read:

```
k1 = $gauss(8,2, name='decayConstant1')$;
```

The Python code will replace the text set off by the dollar signs with a random variable drawn from a Gaussian distribution with mean 8 and standard deviation 2. In the file storing the sample features that gets written, this feature will be named decayConstant1.

This same style is used for both Sim0 and SIM1. The distributions that are allowed, and more usage details, are given in the manual on the SimKern github repository.

If the simulation package to use is MATLAB, the Python package allows a direct process hook via a MATLAB-Python API provided by MathWorks. This speeds up the overall runtime by not requiring the expensive startup time of MATLAB for every run.

Let N be the number of samples we generate for the SIM0 dataset. Let the feature vectors (the parameters that make the samples different from each other) be given by the vectors x_i, $i = 1 \ldots N$. Each x_i vector is a vector of length p, where we are following the standard machine learning notation where p equals the number of features. Let y_i denote the outcome of the simulation, which could be a category (e.g. alive or dead) or a real number. Since we generate these outputs via a simulation, viewing that simulation as a function S^0 we can write $y_i = S^0(x_i)$. The ground truth data generation procedure is depicted in Figure S1.

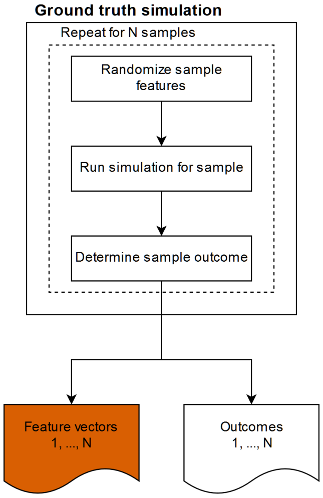

Figure S1: Ground truth data generation procedure, SIM0.

The x data get written to Sim0Genomes.csv and the y data to Sim0Output.csv (the term genome is used since the use case that provides the motivation for this software is

machine learning for biological systems where the feature vector is based on genomics). Separate files, called genome keys, are written out for each sample for use in the SIM1 runs.

1.2 Similarity kernel generation: SIM1

The main document describes the similarity matrix computation. The python software handles writing out and running the individual (i, r) run files, using the .u file as the template. This .u file must reference another file which specifies the parameters from the SIM0 run that make each individual sample i distinct. This file is called genome1_key (the "1" is replaced automatically by the SIM1 python code with the sample number i).

The output of this procedure is the similarity matrix, given in a file called Similarity-Matrixfinal.csv. A similarity matrix is also written after every trial (from the third trial onward; similarity matrices before the third trial are considered not converged yet and so are not written out).

1.2.1 Similarity as measured by closeness of ODE solutions

A typical setting for a SIM1 run will be the simulation of a set of ordinary differential equations (ODEs). In this context, the similarity between population members i and j, for simulation r, can be a measure of how close the overall time dynamics for i are to the time dynamics of j, e.g., represented by the mean squared error over discrete time points. More specifically, assume the ODE simulation contains E different entities (e.g. protein levels), in other words E ODEs. Let us further assume that the simulation program outputs the levels of these entities at a given set of times, $t_1,\ t_2, \ldots, t_k, \ldots t_T$. Let $L_r^i(e, k)$ be the level of ODE entity e at time t_k, for population member i under simulation r. Since the ODE equations may be of different magnitudes, we will normalize each pair being compared by the maximum level that either ever takes over the time course (we are

implicitly assuming the ODEs solutions are always non-negative, this would have to be modified for negative levels). For the pair of samples (i, j) and for entity e in simulation run r, the maximum value M is given by:

$$M(i, j, e, r) = \max[\max_k L_r^i(e, k),\ \max_k L_r^j(e, k)]$$

With these definitions, we can write

$$z(i, j, r) = 1 - \frac{1}{E \cdot T} \sum_{e=1}^{E} \sum_{k=1}^{T} \left(\frac{L_r^i(e, k) - L_r^j(e, k)}{M(i, j, e, r)} \right)^2 \tag{1}$$

Finally, in addition to normalizing the ODE solutions to a maximum value of 1, the user may want to weight the different entities e to express the prior knowledge that some entities are more important for similarity considerations than others. Let $0 \leq w_e \leq 1$ be user-defined weights and then we have:

$$z(i, j, r) = 1 - \frac{1}{E \cdot T} \sum_{e=1}^{E} w_e \sum_{k=1}^{T} \left(\frac{L_r^i(e, k) - L_r^j(e, k)}{M(i, j, e, r)} \right)^2 \tag{2}$$

2 Machine learning details

2.1 Machine learning algorithm comparisons procedure

Machine learning (ML) was conducted in MATLAB (MathWorks, Natick, MA, USA) using the libSVM package for all SVM models (*1*). The python SimKern codebase also provides routines for the machine learning runs. Alg 1. outlines the experimental design to tune the hyperparameters and then estimate performance metrics for each ML algorithm. Although the algorithm initially splits a dataset into three pieces–50% for training, 25% for validation, and 25% for final accuracy assessment–the training subset is further subsampled to assess how accuracy depends on the amount of training data for the various models and machine learning algorithms. The same experiment is repeated for

each dataset. The procedure is outlined below and explained in detail in the subsequent subsections.

```
load data of the ground truth data simulation;
load similarity matrix of the SimKern simulation;
shift and rescale features to [0, 1];
dummy-code categorical features for SVM algorithms;
for repetition i = 1 : 10 do
    randomly sample 50% of all rows as training data (stratify samples if it is a
      classification problem);
    randomly sample 25% of all remaining rows as validation data (stratify samples
      if it is a classification problem);
    assign the remaining rows as test data;
    foreach subsampling percentage s ∈ {s_1, s_2, ..., s_S} do
        randomly subsample s of all training rows as training data (stratify samples
          if it is a classification problem);
        foreach algorithm a ∈ A do
            foreach hyperparameter configuration h_a ∈ H_a do
                train algorithm a with hyperparameter configuration h_a on training
                  data;
                predict outcomes for validation data;
                compute performance metric on validation data predictions;
            end
            select hyperparameter configuration h*_a with best validation performance
              metric;
            select algorithm a trained with hyperparameter configuration h*_a;
            predict outcomes for test data;
            compute performance metric on test data predictions;
        end
    end
end
```

Alg. 1. Experimental design to estimate ML performance (this algorithm is executed independently on each dataset). A is the set of ML algorithms used. s_i subsampling percentages vary by model in order to home in on the most relevant part of the curve which represents accuracy versus amount of training data, see Table S3.

2.1.1 Stratification

For the classification models, the data is split while approximately stratifying for classes. Stratification of classes in training, validation, and test data ensures stability in the estimation process. Consider the case where random sampling led to an unusual distribution of classes in training and validation data. Consequently, the test data would very likely have a class distribution different than the training data. Classifiers not correcting for class imbalance (default RF and default SVMs) that are trained on this training data would perform worse on the test data. Since we want to estimate generalization performance, i.e. performance on the general population with a class distribution estimated by the class distribution in the full dataset, we stratify classes in training and test data.

2.1.2 Hyperparameter tuning

The performance of the studied ML algorithms is dependent on algorithm-specific hyperparameters (HP) whose optimal values for generalization performance are not known *a priori*. HPs are tuned by a grid search: for a selection of values per HP, the algorithm is trained on the training data and evaluated on the validation data for each possible HP combination. The HP combination with the best performance metric in the validation data is selected. Table S2 lists the HPs that are tuned, their ranges, and values on the search grid for each algorithm. Values are partly determined from existing literature or chosen experimentally. HPs not mentioned here are set to default values. Values for SVM parameters are partially taken from (*2*). For RF, the number of trees is fixed at 100. While (*3*) did not limit the number of terminal nodes in a tree, (*4*) provide empirical evidence in favor of tuning. Therefore, we tune the maximal number of splits allowed in a tree. Tuning grid boundaries have been extended manually to reduce the number of cases where the tuning procedure selects HP values on the grid boundaries, which would

suggests that better HP values might be found outside the grid.

Algorithm	HP	Range	Values on grid
linear SVM & SimKern SVM	C	$[0, \infty]$	$\{10^{-12}, 10^{-11}, ..., 10^{12}\}$
	ε	$[0, 1]$	$\{10^{-5}, 10^{-4}, ..., 10^{-1}, 0.25, 0.5, 0.75, 1\}$
RBF SVM	C	$[0, \infty]$	$\{10^{-12}, 10^{-11}, ..., 10^{12}\}$
	γ	$(0, \infty]$	$\{10^{-15}, 10^{-14}, ..., 10^{1}\}$
	ε	$[0, 1]$	$\{10^{-5}, 10^{-4}, ..., 10^{-1}, 0.25, 0.5, 0.75, 1\}$
RF & SimKern RF	*n. feat.*	$[1, \infty]$	$\{1, \lfloor (1+\sqrt{p})/2 \rfloor, \lfloor \sqrt{p} \rfloor, \lfloor (\sqrt{p}+p)/2 \rfloor, p\}$
	n. splits	$[1, (n-1)]$	$\lfloor \{0.05, 0.1, 0.2, 0.3, 0.4, 0.5, 0.75, 1\} n \rfloor$

Table S2: Hyperparameter tuning per algorithm. C is the weight corresponding to training set error in the SVM objective. ε (only used for SVM regression) determines the width of the margin enclosing the separating hyperplane in SVM regression. γ is a parameter of the RBF kernel $K(x, y) = \exp(-\gamma||x - y||^2)$. *n. feat.* is the number of randomly sampled features compared at each split in a tree. *n. splits* is the maximal number of splits per tree, grid values exceeding the interval $[1, (n-1)]$ are truncated to the boundary. n is the number of training samples, p is the number of features.

Model	s_1	s_2	s_3	s_4	s_5
Radiation	5%	10%	25%	50%	100%
Flowering	5%	10%	30%	60%	100%
Boolean	2.5%	5%	10%	20%	100%
Network	4%	7%	10%	13%	16%

Table S3: Subsampling training percentages per model.

2.2 Machine learning algorithms

We compare standard machine learning algorithms that use the ground truth feature vectors (Standard ML algorithms) to ML algorithms that use the SimKern kernel matrix (*SimKern* ML algorithms), see Figure S2. For the Standard ML learning, we utilize three established ML algorithms: linear SVM (*5*), radial basis function (RBF) SVM, and random forest (RF) (*3*). For SimKern learning, we use SVM with the similarity matrix

as a custom kernel (note that for the SVM algorithm the kernel matrix, also known as the Gram matrix, has to be symmetric positive definite, which in all of our models is the case, and indeed is required by the libSVM software) and the random forest algorithm with the similarity matrix as the feature matrix input (*6*). This random forest, called SimKern RF, classifies new samples according to their similarities with training samples. Additionally, we compute nearest neighbor predictions to compare to the more advanced machine learning algorithms. For the Standard ML case, we use a 1-NN algorithm on the SIM0 feature vector. For the SimKern case, we use the label of the most similar distinct training sample according to the similarity matrix. We label this approach *SimKern NN*.

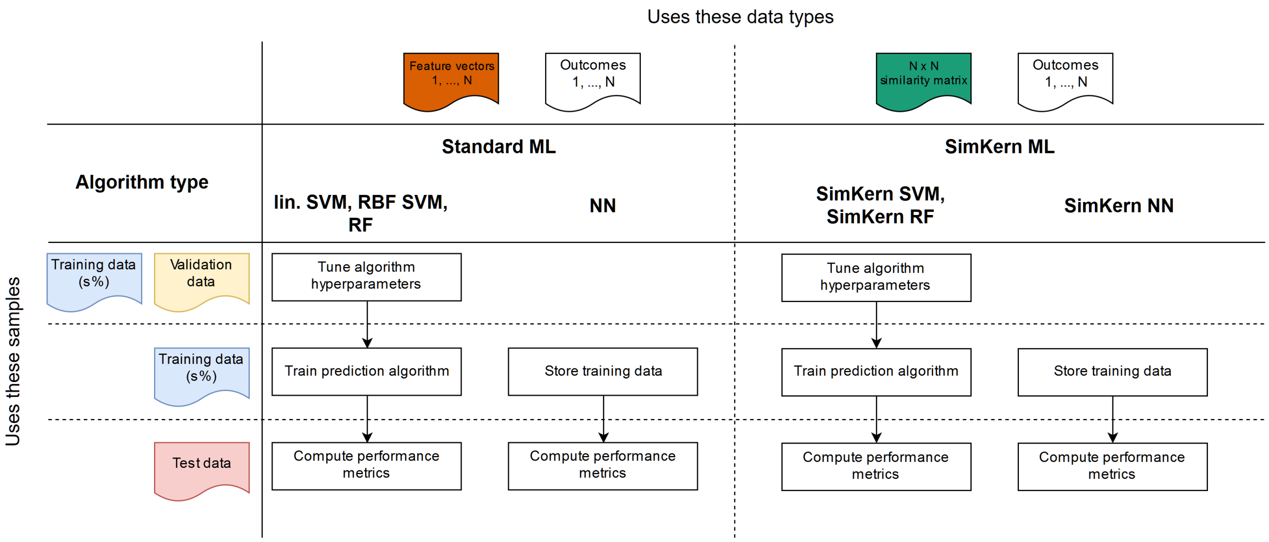

Figure S2: An overview of the data handling procedures for the various machine learning algorithms used. SVM=support vector machine, RBF=radial basis function, ML=machine learning, NN=nearest neighbors, RF=random forest.

3 Model descriptions

Table S4 gives a summary of the machine learning problem sizes, number of features, and other attributes, for the four models.

Model	Class 1	Class 2	Class 3	Class 4	n	p	R
Radiation	27.5%	23%	44.2%	5.3%	1000	39	20
Flowering	-	-	-	-	500	35*	5
Boolean	62.5%	9.3%	28.2%	-	1000	37	20
Network	61.6%	18.2%	20.2%	-	500	12	10

Table S4: Numerical information for the four models. Class distribution per model for the ground truth (SIM0) dataset. Note that the Flowering model has continuous outcomes (i.e. flowering time) and the Boolean and Network models have only three classes. Classes (in order 1, 2, 3, 4) for the Radiation model are apoptosis, repaired and cycling, mitotic catastrophe, and quiescence. For the Boolean cancer model they are apoptosis, metastasis, and other. For the Network model they are simply which of the exit arcs the optimal solution flows through. n is the number of samples generated for the SIM0 ground truth dataset, p is the number of features in the ground truth dataset, and R is the number of trials run in the SimKern step. *For the flowering model one of the features is a categorical variable of 19 classes, representing 19 different mutational states. Thus if one-hot encoded this would lead to an additional 19 features.

3.1 Radiation model

The radiation model is built up as four connected modules. We opt to not simulate the cell cycle and instead focus on the chain of events that happens after radiation damages a cell's DNA: DNA repair (modeled at a high level), p53-based transcription factor control, cell cycle arrest, and apoptosis, see Figure S3. Although highly simplified, this model recapitulates the idea that the inter-connected dynamics of these processes determine cell fate after radiation damage.

Tuning this model to reflect the behavior of an actual cell line is very large task, and probably not possible in any realistic way, since the genes (proteins) chosen to be in the model are but a small subset of the proteins involved in a DNA repair and cell cycle control cascade. However, even without validated rate constants chosen, the model provides a numerical instance of a complex system, based on known biology, where different modules (biochemical processes) are involved in determining the fate of a cell subject to an external

stimulus. We hand tuned the parameters of the base model. There are many parameters to choose from, and our choices were from manual explorations which led to a set of parameters that led to diverse system behavior (some samples ending in apoptosis, others in cell cycle arrest, etc.).

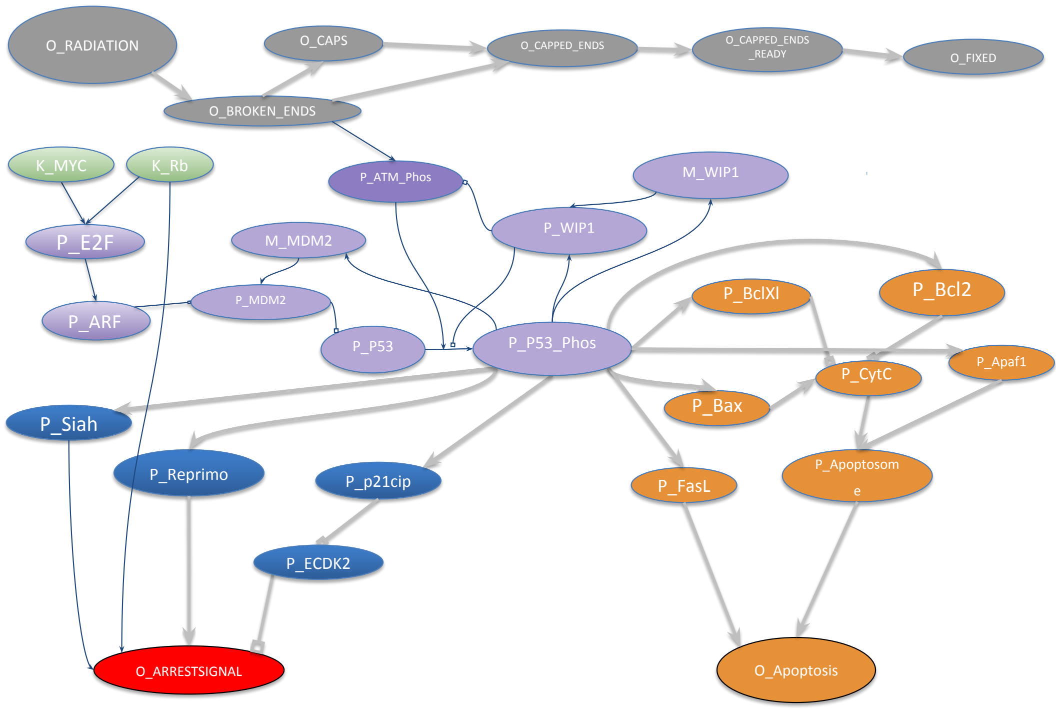

Figure S3: A model of entities and processes involved in cell fate decision following radiation. The gray nodes depict the process of DNA breakage and repair. DNA breaks send signals via ATM to the p53-MDM2-ARF module (purple), which in turn sends both apoptotic signals (orange) and cell cycle arrest signals (blue). The cancer genes MYC and Rb (green) are modeled as fixed parameters rather than time varying entities. The first letters of each oval have the following meanings: P = protein, M = mRNA, K = rate constant, O = other. Phos stands for phosphorylated.

The p53-MDM2 transcription regulatory control circuit comes from (*7*). We use the single compartment version of the model, where the specific location of molecules (nucleus versus cytoplasm) is ignored. Radiation damage affects this circuit via the ATM kinase

pathway, which increases the phosphorylation and hence stability of p53. p53 then goes on to be a transcription factor for apoptosis and cell cycle arrest genes.

Cell specific alterations (mutations, amplifications, deletions) for MYC, RB1, and p53 interact to influence how the p53-MDM2 circuit behaves, which in turn affects the behavior of the downstream processes of cell cycle arrest and apoptosis. The number of cell cycle controls in an eukaryotic cell is large. Rather than attempting to model most of them, we choose a few overlapping controls to create a model that creates a challenging machine learning problem.

Apoptosis is modeled as the competition between pro-apoptotic (BAX, FasL) and anti-apoptotic proteins (BCL-2, BCL-xl). Apoptosis occurs if the apoptosome is formed (a combination of cytochrome c and APAF-1, which together release caspases from the mitochondrial membrane) or via the extrinsic Fas/FasL pathway.

The detailed mathematical model is given next. In the ODE equations as written below, we use a generic "k" for ODE constants, to reduce clutter. For the full details, we refer the reader to the MATLAB code.

Phosphorylated nuclear p53 protein tetramerizes to form its active transcription factor state. For convenience we define the p53 tetramerized term as:

$$p53tt = (MUT_{p53} * pP53NucPhos^4) \tag{3}$$

The mutation coefficient MUT_{p53} is a uniform random variable between 0 and 1, reflecting the idea that there are a large number of p53 mutations that potentially affect the tetramerization in varying ways.

The full ODE model is given here:

$$\dot{oRadiation} = -k * oRadiation$$

$$\dot{oBrokenEnds} = k * oRadiation - k * oBrokenEnds * oCaps$$

$$\dot{oCaps} = \min\left((k * oBrokenEnds), k_5\right) - k * oBrokenEnds * oCaps - k * oCaps$$

$$\dot{oCappedEnds} = k * oBrokenEnds * oCaps - k * oCappedEnds$$

$$\dot{oCappedEndsReady} = k * oCappedEnds - k * oCappedEndsReady$$

$$\dot{oFixed} = k * oCappedEndsReady$$

$$\dot{pP53Nuc} = k + k * pWIP1Nuc * \frac{pP53NucPhos}{+pP53NucPhos} - k * pMDM2Nuc * \frac{pP53Nuc}{k * pP53Nuc} - k * pATMNucPhos \frac{pP53Nuc}{k * pP53Nuc} - k * pP53Nuc$$

$$\dot{pMDM2Nuc} = k * mMDM2Nuc - pMDM2NUC - MUT_{arf} * k * pARF * pMDM2Nuc$$

$$\dot{mMDM2Nuc} = k + k * \frac{p53tt}{k^4 + p53tt} - k * mMDM2Nuc - k * mMDM2Nuc$$

$$\dot{pP53NucPhos} = k * pATMNucPhos * \frac{pP53Nuc}{k * pP53Nuc} - k * pWIP1Nuc * \frac{k * pP53NucPhos}{k + pP53NucPhos}$$

$$\dot{pWIP1Nuc} = k * mWIP1Nuc - kpWIP1Nuc$$

$$\dot{mWIP1Nuc} = k + k * \frac{p53tt}{k^4 + p53tt} - k * mWIP1Nuc - k * mWIP1Nuc$$

$$\dot{pATMNucPhos} = 2 * k * oBrokenEnds * \frac{\frac{k - pATMNucPhos}{2}}{k + \frac{k - pATMNucPhos}{2}} - 2 * k * pWIP1Nuc * \frac{ATMNucPhos^2}{k + pATMNucPhos^2}$$

$$\dot{pBcl2} = k * \frac{p53tt}{k + p53tt} - k * pBcl2$$

$$\dot{pBclXl} = k * \frac{p53tt}{k + p53tt} - k * pBclXl$$

$$\dot{pFasL} = k * \frac{p53tt}{k + p53tt} - k * pFasL$$

$$\dot{pBax} = MUT_{Bax} * \left(k * \frac{p53tt}{k + p53tt} - k * pBax \right)$$

$$\dot{pApaf1} = MUT_{Apaf1} * \left(k * \frac{p53tt}{k + p53tt} - k * pApaf1 \right)$$

$$\dot{pCytC} = k * \frac{1}{1 + e^{-k*pBax-k}} * k * (1 - \frac{1}{1 + e^{-k*pBcl2-k}}) * k * (1 - \frac{1}{1 + e^{-k*pBclXl2-k}}) - kpCytC - k * pApaf1 * pCytC^7$$

$$\dot{pApoptosome} = k * pApaf1 * pCytC^7 - k * pApoptosome$$

$$\dot{oApoptosis} = k * pFasL + k * pApoptosome - k * oApoptosis$$

$$\dot{pE2F} = MUT_{Rb} * MUT_{myc} - kpE2F$$

$$\dot{pARF} = MUT_{arf} \left(k1 * \frac{pE2F}{k + pE2F} - k2 * pARF - k * pARF * pMDM2Nuc \right)$$

$$\dot{pP21cip} = k * \frac{p53tt}{k + p53tt} - k * pP21cip$$

$$\dot{pECDK2} = k - \frac{k * pP21cip}{k + pP21cip} - (k * pECDK2)$$

$$\dot{pSiah} = MUT_{Siah} \left(\frac{k * p53tt}{k + p53tt} - k * pSiah \right)$$

$$\dot{pReprimo} = MUT_{Reprimo} \left(\frac{k * p53tt}{k + p53tt} - k * pReprimo \right)$$

$$\dot{oArrestsignal} = (see\ below)$$

The initial condition of the system is an externally applied radiation dose modeled by setting $oRadiation(0) = 1$ followed by an exponential decay. The only other non-zero initial condition is for ECDK2 since at time 0 we assume that there are no brakes on the cell cycle.

3.1.1 Additional modeling notes

Cells have many mechanisms to control cell growth and division. We choose to model just a few, and in a simplified manner, to get the flavor of the complexity. We split the control into two cases, one where the Rb gene is functioning ($Rb = 1$) and one where the Rb gene is impaired ($Rb = 0$). For the $Rb = 0$ case, a way to arrest cell growth is via the SIAH or Reprimo gene pathways. SIAH and Reprimo are activated by a functioning p53 danger signal pathway, and we model their effect on the arrest signal as additive. Thus:

Case $Rb = 0$:

$$oArrestsignal = \frac{1}{1 + e^{ka1*(x(Siah)+x(Reprimo)-ka2)}}; \tag{4}$$

We take the derivative of this to embed it into the ODE set.

For $Rb = 1$, the Rb controls are working correctly. In that case, low levels of the Cyclin E/CDK2 complex (ECDK2) will arrest the cell cycle, independently of SIAH and Reprimo levels. On the other hand, high levels of ECDK2 mean that the cell can pass through the G1-S transition, but SIAH or Reprimo might still stop it. We model this as a convex combination for the arrest signal:

Case $Rb = 1$:

$$oArrestsignal = \lambda_{low} * 1 + (1 - \lambda_{low})\frac{1}{1 + e^{ka1*(x(Siah)+x(Reprimo)-ka2)}}; \tag{5}$$

where $\lambda_{low} = 1 - (ECDK2/ECDK2_{max})$, where $ECDK2_{max}$ is the maximum level that ECDK2 can attain. We differentiate this as above.

The final classification (into one of four states: 1, 2, 3, or 4) for the ground truth simulation uses the following rules, based on the levels at the end of the simulation:

```
If Apoptosis >= 0.8:                     1 (apoptosis)
Else
```

```
If FIXED > 0.9 and ARREST < .5:       2 (repaired and cycling)
Else if FIXED <= 0.9 and ARREST < .5: 3 (not repaired, and cycling
                                        i.e. mitotic catastrophe)
Else                                  4 (quiescence)
```

For details on mutations and parameter changes used for ground truth dataset and the kernel dataset, see the MATLAB input files.

3.2 Flowering model

The flowering model is taken directly from (*8*). The outcome that we build a prediction model for is flowering time, which, as in the original paper, is taken to be the time at which the protein AP1 exceeds a given threshold. The ODE model is simulated using MATLAB. The flowering model represents an isolated genetic circuit in multi-cellular eukaryote, and therefore as a model is a distant cousin–but a relevant one–to the vastly complicated genetic circuitry of human cancer cells.

3.3 Boolean cancer model

The Boolean cancer model is taken from (*9*). We converted their GinSIM model into BoolNet format, which is a package in R. The authors provide an original model as well as a modular reduction. In the SimKern simulation we use the modular reduction, which represents a limited understanding of the model, further perturbed by uncertainties of how to map the feature data into this reduced model. The model output for both the ground truth dataset and the SimKern runs are based on the steady state vectors found by simulating the network for the given initial conditions. Let $ss(n)$ denote the steady state value for node n. If the steady state is a fixed steady state, $ss(n)$ will be a single value, either 0 or 1. If the steady state is a cycle, then $ss(n)$ will be a binary vector

of length equal to the cycle length. For the classification, we rely on two compartments in particular: n = Apoptosis and n = Metastasis. We classify the outputs into three categories using the following logic.

```
If all(ss(Apoptosis)) = 1:         1 (apoptosis)
Else if all(ss(Metastasis)) = 1:   2 (metastasis)
Else                               3 (other)
```

For details about the meaning of this model we refer the readers to the original publication (*9*). In the present work, it is sufficient to view this model as an instance of a discrete complex system.

3.4 Network flow optimization model

We wrote a random network generation routine in MATLAB which generates a layer-wise directed graph. The user specifies the number of nodes for each layer and probabilities for adding a connecting arc between the nodes of two layers. We also add arcs between non-adjacent layers with a small probability. We ran this routine once to create a single network for all the samples in the dataset, shown in Figure S2. We generate random numbers for the cost for these arcs. This represents the base network from which all the samples of SIM0 are built. Unique samples are created by varying the weights of 12 of the 80 arcs, the bold arcs in Figure S2. The outcome of the simulation is a classification, 1, 2, or 3, representing which of the last three arcs the optimal flow passes through (linear network flow optimization theory guarantees that there exists an optimal solution with all the flow through one of the exit arcs, and that such a solution will be returned by simplex-based methods (*10*)).

We run two versions of SIM1, a *less noisy* model (with fewer perturbed, less noisy arc costs) and a *noisier* model (with larger number and higher magnitude of perturbed arc

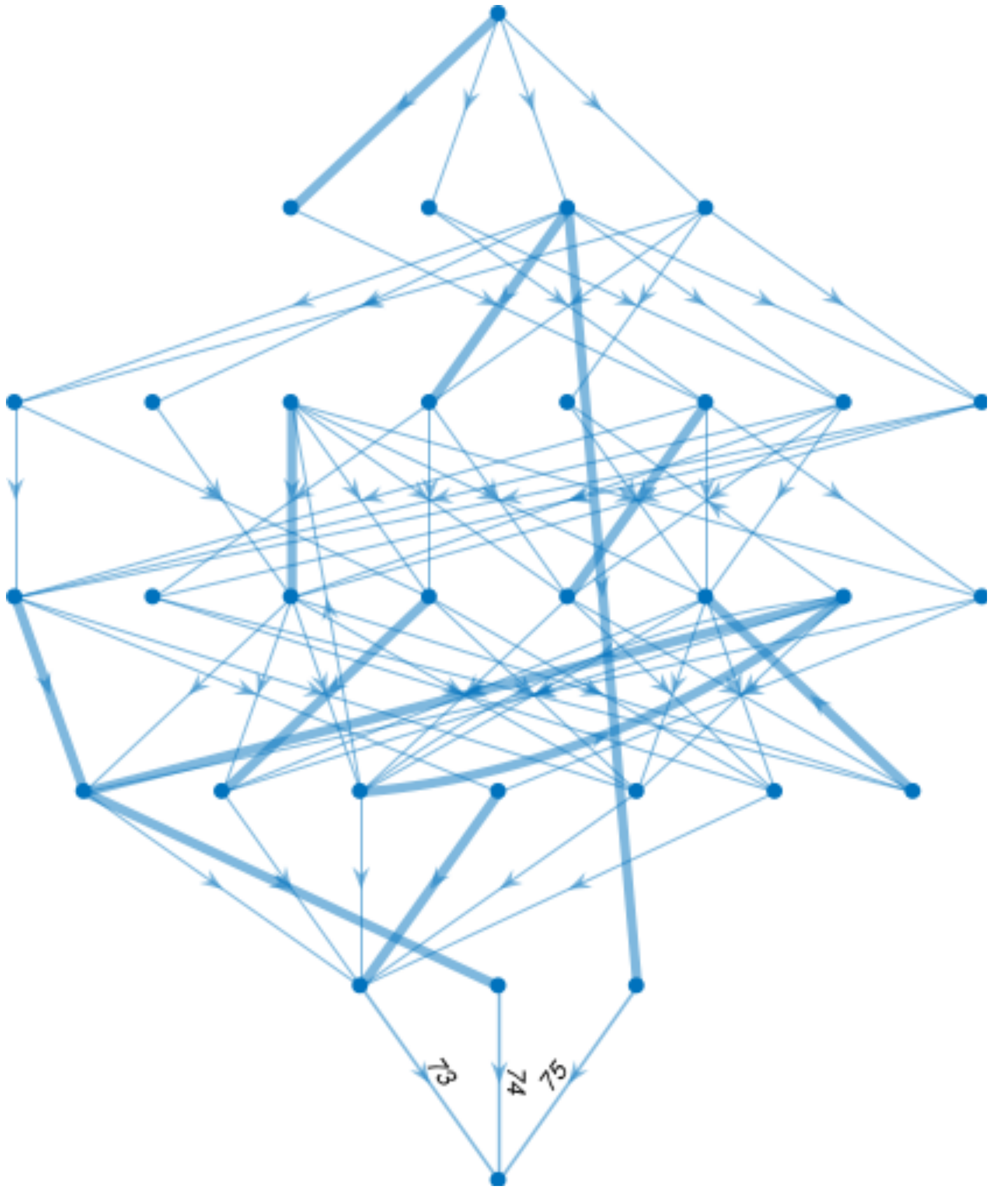

Figure S4: Network flow directed graph. The bold lines are the arcs with variable costs in the ground truth simulation. The unit flow that enters the network at the uppermost node will exit through one of the labeled arcs at the bottom, which creates a classification problem.

costs). For the *less noisy* models we assume the arc costs of the 12 SIM0 variable arcs are not known with certainty: they are scaled by a uniformly distributed random variable between 0.1 and 1.9. We also perturb every arc in the second layer by a uniform variable

from 0.5 to 1.5. For the *noisier* model we additionally perturb the arc costs of the third layer (uniform 0.5 to 1.5) as well as a large perturbation, uniform between 9 and 10, of the third arc, which otherwise always takes the flow because of its otherwise low arc cost (see Figure S4).

4 Supplementary results

The flowering model, Figure S5, displays a typical "good kernel" result where the SimKern methods dominate the no-prior-knowledge methods throughout, but especially for small training set sizes. Similarity based NN is competitive with the more sophisticated similarity SVM and RF, but exhibits slightly more variance. The success of the SimKern methods indicates that the space induced by the similarity kernel is well behaved and the classes are easily separable with this kernel.

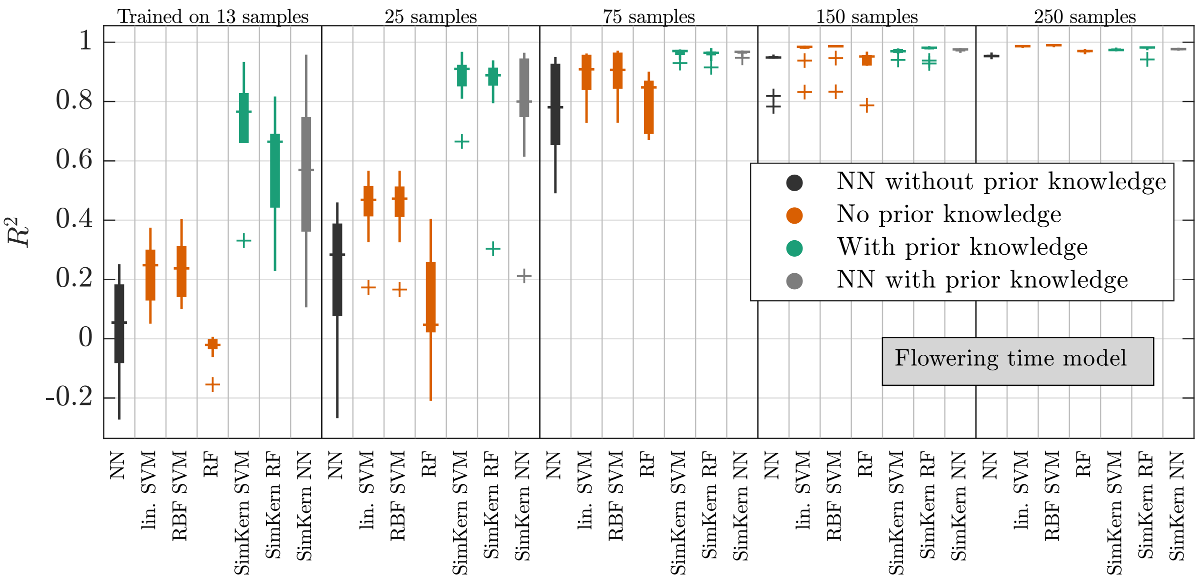

Figure S5: Machine learning results for the flowering model. NN = nearest neighbor, RF = random forest, SVM = support vector machine, RBF = radial basis function. R^2 is the coefficient of determination.

With the network flow model, we demonstrate the obvious but important result that if the SimKern simulation is farther from the ground truth simulation due to additional noise, the SimKern learning will be worse. The kernel based on a *less noisy* SimKern simulation, Figure S6, displays dominance throughout whereas the kernel based on a *noisier* SimKern simulation, Figure S7, is overtaken by the standard RF already by 18 training samples. We also used vector-based outputs from the SIM1 simulations, where the flow through every arc was used to compute similarity scores. The results were not fundamentally different so here we display results from only the scalar based SIM1 output.

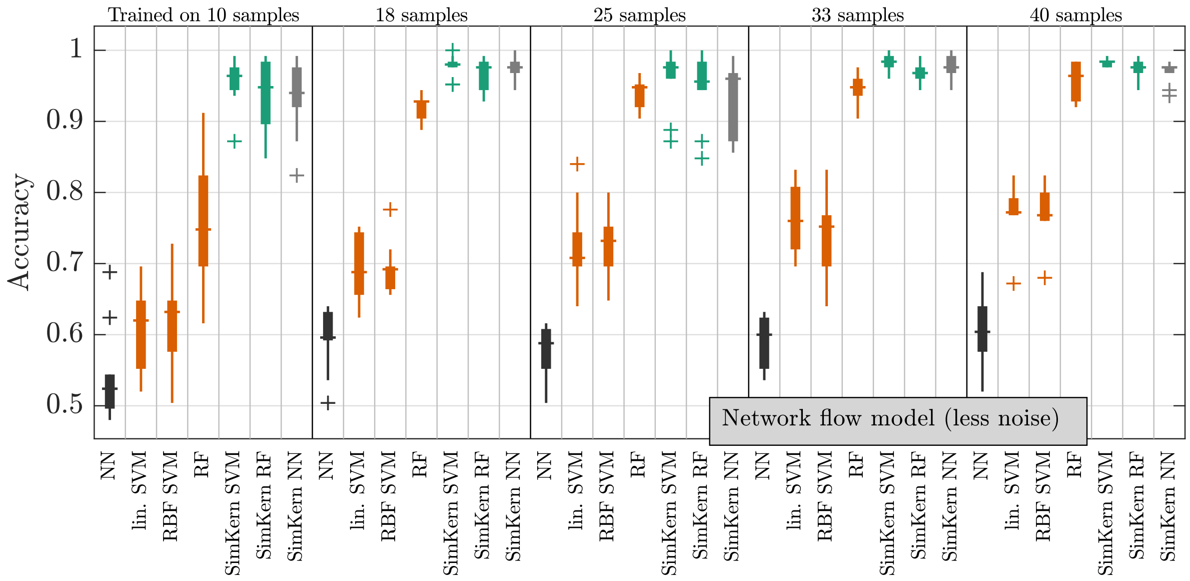

Figure S6: Machine learning results for the network flow optimization model for the *less noise* case. NN = nearest neighbor, RF = random forest, SVM = support vector machine, RBF = radial basis function.

The SimKern idea is effective provided that the simulations correctly judge the similarity between two samples, but the SimKern simulations need not themselves make correct predictions (in fact, the raw output of the SimKern simulations need not be the same type of output as we are trying to predict). To illustrate this, we examine the first 13

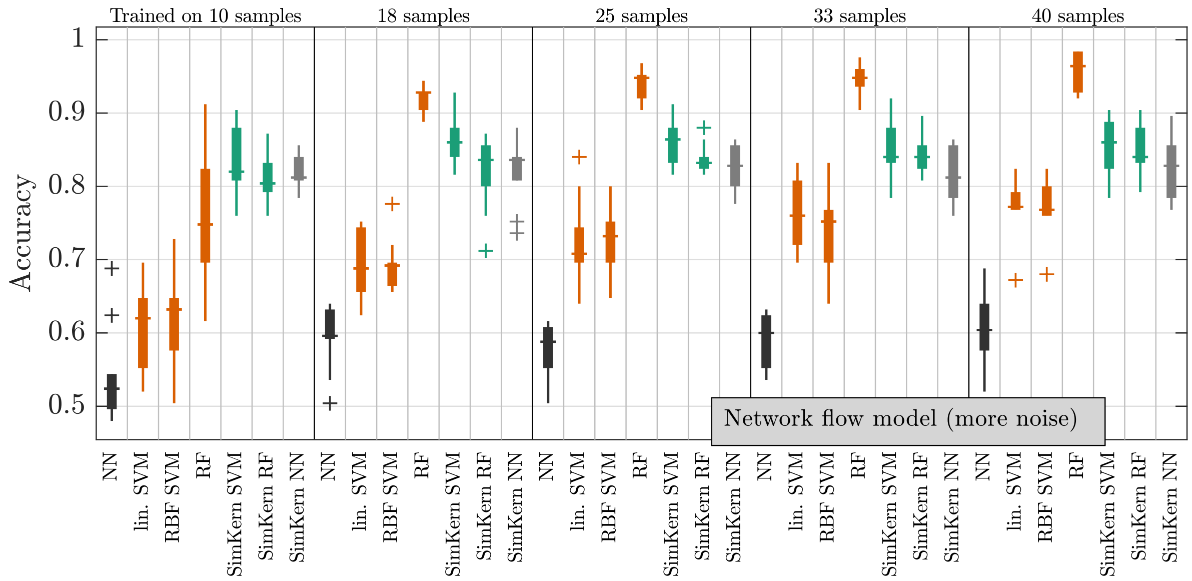

Figure S7: Machine learning results for the network flow optimization model for the *more noise* case. NN = nearest neighbor, RF = random forest, SVM = support vector machine, RBF = radial basis function.

samples from the dataset for the network (lower quality) model, see Figure S8. Samples 2 and 11, which both are classified as 3s in the ground truth dataset, are given a high similarity score because they behave similarly for most of the 10 trials, even though in only one of those trials (trial 6) are they actually classified correctly.

Each model displays two nearest neighbor (NN) algorithm learning results: the default method which is Euclidean distance in feature space, and the kernelized method which uses the simulation based similarity scores for the distance computation. Consistently, the kernel based NN methods dominates over standard NN, which implies the power of a custom similarity measure. The difference between either of these NN methods and the SVMs display the power of better machine learning algorithms: rather than classifying a new sample based on which training sample it is closest to, SVMs factor in the distance to many of the training samples. In some cases (Figure 3, main document: the radiation

model with the higher quality kernel, and Figure 4 main document or Figure S5: the flowering model) we see that a good similarity score is ultimately good enough and more advanced machine learning algorithms do not offer much improvement over the kernelized NN.

Sample →	1	2	3	4	5	6	7	8	9	10	11	12	13
Trial 1 outcome	2	2	2	2	2	1	2	2	2	2	1	1	2
Trial 2 outcome	1	1	1	1	1	1	1	3	1	1	1	1	1
Trial 3 outcome	1	1	1	1	2	1	2	2	1	2	1	1	1
Trial 4 outcome	1	1	1	1	1	1	1	3	1	1	1	1	1
Trial 5 outcome	2	2	2	2	2	2	2	2	2	2	2	2	2
Trial 6 outcome	1	3	1	3	1	3	2	3	1	1	3	1	3
Trial 7 outcome	2	2	2	2	2	2	2	2	2	2	2	2	2
Trial 8 outcome	1	1	1	1	1	1	2	3	1	1	1	1	1
Trial 9 outcome	1	1	1	1	2	1	2	2	1	2	1	1	1
Trial 10 outcome	1	2	2	1	2	1	2	2	2	2	1	1	2
Ground truth	1	3	1	3	1	3	2	3	1	1	3	1	1

Figure S8: SIM1 results for the first 13 samples from the network (lower quality) dataset, for all ten trials and also showing in the bottom yellow row the ground truth (SIM0) result. We have highlighted samples 2 and 11. These samples are both 3s in the ground truth set, but in the $R = 10$ SimKern (SIM1) trials they get correctly classified only once. However, they are given a high similarity score since they behave the same for most of the trials. We use this to highlight the idea that it is sufficient to correctly judge sample similarity; accurate class prediction is not necessary.

As an additional way to compare machine learning results in the case of regression (the flowering model), Figure S9 plots the predicted flowering times versus the actual flowering times. With additional training samples (13 to 25), linear SVM and, even more so, SimKern SVM improve their predictions for samples with a flowering time < 6. After training on additional data, one observes a small additional downward bias in linear SVM predictions for samples with a flowering time > 10. Both algorithms, however, achieve an

R^2 improvement by 0.19 (linear SVM) and 0.31 (SimKern SVM).

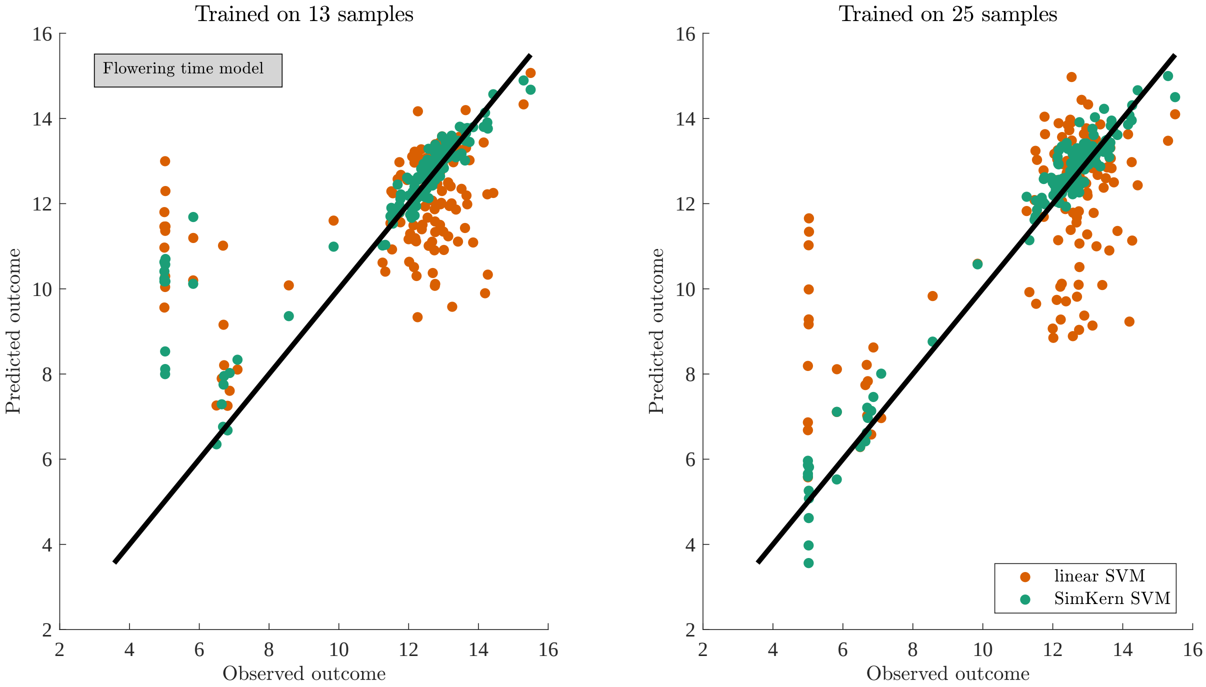

Figure S9: Observed and predicted test set values after training on 13 (left) and 25 (right) samples for the flowering time model. Results for linear SVM and SimKern SVM are in orange and green, respectively. Left: R^2 equals 0.27 and 0.66 for linear SVM and SimKern SVM, respectively. Right: R^2 equals 0.46 and 0.97 for linear SVM and SimKern SVM, respectively.

5 A word on the "kernel trick"

Kernel methods are often touted in the literature as a cure-all for the problem of overly high dimensional samples: by kernelizing the data, the high dimensionality goes away. In fact, kernelizing data does not so cleanly solve this problem since there are many ways to make a kernel. Only when considering highly restricted kernel classes such as linear kernels or RBF kernels, without any feature weighting or feature selection, does the kernel trick simplify the search for a good machine learning approach. But in general, we do not

know how to build a good kernel (that is, how to judge similarity between two samples in a way that is most effective for our machine learning problem). We propose herein to distill expert knowledge of a domain into simulations that use the high dimensional features, which pre-supposes quite detailed knowledge of the system. If such detailed knowledge is not available, the number of ways to turn a large feature set into a kernel is unmanageable (consider combinatoric calculations for example of selecting 200 genes out of 20000 to test all sets of 200 genes). We state this here as a word of caution: the kernel approach can be very useful but it requires obtaining a good kernel, and there is no general recipe for that.

Clearly for the SimKern approach to work, the simulations used to generate the kernel have to be "good", but unfortunately, it does not seem possible to be more quantitative than that for general cases. We explored the issue by demonstrating that as we veer away from high quality simulations, the machine learning using the custom kernel does worse (see e.g. Figures S6 and S7), but it will always be a data- and problem-specific analysis to see if a proposed kernel is useful.

References and Notes

1. Chih-Chung Chang and Chih-Jen Lin. LIBSVM: A library for support vector machines. *ACM Transactions on Intelligent Systems and Technology*, 2:27:1–27:27, 2011. Software available at `http://www.csie.ntu.edu.tw/~cjlin/libsvm`.

2. Asa Ben-Hur and Jason Weston. A users guide to support vector machines. *Data mining techniques for the life sciences*, pages 223–239, 2010.

3. Leo Breiman. Random forests. *Machine learning*, 45(1):5–32, 2001.

4. Roxane Duroux and Erwan Scornet. Impact of subsampling and pruning on random forests. *arXiv preprint arXiv:1603.04261*, 2016.

5. Corinna Cortes and Vladimir Vapnik. Support-vector networks. *Machine learning*, 20(3):273–297, 1995.

6. Saket Sathe and Charu C Aggarwal. Similarity forests. In *Proceedings of the 23rd ACM SIGKDD International Conference on Knowledge Discovery and Data Mining*, pages 395–403. ACM, 2017.

7. Ján Eliaš, Luna Dimitrio, Jean Clairambault, and Roberto Natalini. The p53 protein and its molecular network: modelling a missing link between dna damage and cell fate. *Biochimica et Biophysica Acta (BBA)-Proteins and Proteomics*, 1844(1):232–247, 2014.

8. Felipe Leal Valentim, Simon van Mourik, David Posé, Min C Kim, Markus Schmid, Roeland CHJ van Ham, Marco Busscher, Gabino F Sanchez-Perez, Jaap Molenaar, Gerco C Angenent, et al. A quantitative and dynamic model of the arabidopsis flowering time gene regulatory network. *PloS one*, 10(2):e0116973, 2015.

9. David PA Cohen, Loredana Martignetti, Sylvie Robine, Emmanuel Barillot, Andrei Zinovyev, and Laurence Calzone. Mathematical modelling of molecular pathways enabling tumour cell invasion and migration. *PLoS computational biology*, 11(11):e1004571, 2015.

10. D. Bertsimas and J. Tsitsiklis. *Introduction to linear optimization.* Athena Scientific, 1997.
